# Cardiovascular changes, laboratory findings and pain scores in calves undergoing ultrasonography-guided bilateral rectus sheath block before herniorrhaphy: a prospective randomized clinical trial

**DOI:** 10.1186/s12917-023-03754-6

**Published:** 2023-10-05

**Authors:** Maria Chiara Alterisio, Fabiana Micieli, Giovanni Della Valle, Ludovica Chiavaccini, Giancarlo Vesce, Paolo Ciaramella, Jacopo Guccione

**Affiliations:** 1 Department of Veterinary Medicine and Animal Productions, University of Napoli Federico II, Via Delpino 1, Napoli, 80137 Italy; 2 Department of Comparative, Anesthesiology and Pain Management, Department of Comparative, Diagnostic, and Population Medicine, College of Veterinary Medicine, University of Florida, 2015 SW 16th Ave, Gainesville, 32608 FL USA

**Keywords:** Holter monitoring, Health, Welfare, Rectus sheath block, Umbilical hernia

## Abstract

**Background:**

The study aimed to assess the clinical utility of a multiparametric approach to measure the impact of bilateral ultrasound-guided rectus sheath blocks (RSB) on heart rate, serum cortisol concentrations, and pain in calves undergoing herniorraphy. Fourteen calves were randomly assigned to receive either the RSB (RSB group, *n* = 7, injected with 0.3 mL/kg of bupivacaine 0.25% and 0.15 µg/kg of dexmedetomidine per side) or a sham injection (CG group, *n* = 7, injected with an equivalent volume of sterile saline solution). Monitoring included (i) continuous Holter recording from 120 min pre-surgery to 120 min post-surgery; (ii) serum cortisol concentration (SC) at -150 min pre-surgery (baseline), induction time, skin incision, end of surgical procedure (EP-t), and then 30 min, 45 min, 60 min, 120 min, 360 min after recovery; (iii) UNESP-Botucatu pain evaluation at -150 min pre-surgery and 30 min, 45 min, 60 min, 120 min, 240 min, 360 min after recovery.

**Results:**

A significant difference in the heart rate was observed within the RSB group, in the time frame between 120 min to induction compared to the time frame between induction to EP-t period. The SC concentration was significantly higher in the CG at the skin incision. Calves in the RSB group recorded significantly lower pain scores at 45 min, 60 min, 120 and 240 min after recovery.

**Conclusions:**

The study demonstrated that monitoring heart rate and serum cortisol concentrations effectively quantified the effects of RSB during surgery. At the same time, the UNESP-Botucatu pain scale identified effects post-surgery when the calves regained consciousness. Overall, ultrasound-guided RSB appeared to enhance the well-being of calves undergoing herniorrhaphy.

**Supplementary Information:**

The online version contains supplementary material available at 10.1186/s12917-023-03754-6.

## Background

The increasing attention to the health and welfare of animals intended for human consumption promoted the development of novel and targeted therapeutic strategies to reduce discomfort and pain [1]. Many clinical studies investigated how to prevent injuries or diseases in these animals, but only a few explored the potential adverse effects of therapeutic procedures [1–3]. Although choosing appropriate anesthetic or analgesic drugs is crucial, the options in bovine medicine are often limited (e.g., by economic considerations, national legislation, and skills) [4, 5]; so, the selection of anesthetic and analgesic protocols primarily result from the need to strike a balance between these various factors [4–6].

Different systems can be used to assess acute postoperative pain and stress in ruminants. The measurement of the heart rate and rhythm using a Holter monitor, the evaluation of the serum cortisol concentration (SC), and the application of the unidimensional UNESP-Botucatu pain scale have all been considered valid and reliable methods. Digital Holter monitoring records heart rate (HR) changes and dysrhythmias due to discomfort and stress [7]. Changes in SC concentrations can assess the negative effects of improper handling, pathologies, or surgeries [8–10]. Finally, the UNESP-Botucatu pain scale can be used to evaluate acute postoperative pain and to define the degree of analgesia in livestock, with demonstrated reliability and excellent internal consistency [11–13]. This is especially important in cattle field medicine, where other diagnostic methods may be impractical due to long processing time, specific skills, and high economic costs (e.g., precision farming devices) [14, 15]. The diagnostic merits of each system are well documented; therefore, a simultaneous multiparametric-based approach should represent a superior strategy to quantify discomfort and pain in ruminants [16].

Considering the premises, the study aimed to describe a multiparametric approach to measure the effects of a novel locoregional anesthetic technique in calves undergoing herniorrhaphy, one of the most common surgeries performed in the field in this species [6, 17]. The study hypothesized that a multiparametric approach could discriminate between animals receiving and not- receiving an ultrasound-guided rectus sheath block (RSB) as an analgesic strategy for herniorrhaphy. This hypothesis was verified by simultaneously evaluating the impact on the heart rate and rhythm, serum cortisol concentrations, and animal behavior after herniorrhaphy.

## Results

### General

At the end of the recruitment period, seven calves were enrolled in the RSB group and seven in the control group (CG).

Calves aged between 3 and 5 months; they were six males and eight females, belonging to three breeds (Holstein-Friesian, Italian Braun, crossbreed), and weighted between 70 and 190 kg.

None of the surgeries required more than 20 min from skin incision to end of surgical procedure, and none of the animals enrolled was excluded from the study due to clinical complications arising during the entire monitoring period (24 h pre- to 24 h post-herniorrhaphy). All calves were classified in good clinical conditions 24 h after surgery, without fever or other signs of distress; nonetheless, they all showed edema at the surgical site. The farmers reported no clinical complications or recurrence in the 15 days following surgery.

### Heart rate and rhythm

The mean number of heartbeats recorded during the 240-minute Holter recording was 23,040 and 23,520 in the RSB and CG groups, respectively. Specific HR observed in the RSB group and CG at different time intervals are reported in Table 1.

**Table 1:** Average heart rate values and type of cardiac arrhythmias (number of animals affected in brackets) detected in calves receiving ultrasound-guided rectal sheath block (RSB) or a sham injection (control group, CG) at different time intervals (Int)

	***Heart rate***															
	***Int-1***		***Int-2***		***Int-3***		***Int-4***		***Int-5***		***Int-6***		***Int-7***		***Int-8***	
	*bpm*	±*SD*	*bpm*	±*SD*	*bpm*	±*SD*	*bpm*	±*SD*	*bpm*	±*SD*	*bpm*	±*SD*	*bpm*	±*SD*	*bpm*	±*SD*
***RSB***	95.8	19.4	99.9^**a**^	21.0	90.7^**b**^	22.0	94.6	18.6	90.4	18.3	89.8	18.2	92.3	17.4	94.6	18.6
***CG***	97.7	20.6	99.2	19.0	94.6	18.6	98.6	20.5	92.0	18.4	94.5	20.0	95.0	18.2	98.6	20.5
	**Cardiac arrythmias*(n of calves affected)***															
	***Int-1***		***Int-2***		***Int-3***		***Int-4***		***Int-5***		***Int-6***		***Int-7***		***Int-8***	
	*Type(n)*		*Type(n)*		*Type(n)*		*Type(n)*		*Type(n)*		*Type(n)*		*Type(n)*		*Type(n)*	
***RSB***	/		RA*(2)* PVC*(1)* AVB*(4)*		AVB*(1)*		RA *(1)* AVB*(3)*		/		/		/		/	
***CG***	/		RA*(3)* ST*(1)* AVB*(3)*		AVB(2)		TS*(1)* RA*(1)* AVB*(1)* PVC *(1)*		/		/		/		/	

*Bpm* beats per minute, *SD* standard deviation, *Int-1* -120min pre-surgery to +120min post-surgery, *Int-2* -120min pre-surgery to beginning of IND-t, *Int-3* beginning of IND-t to EP-t, *Int-4* EP-t to +120min post-surgery, *Int-5* EP-t to +15min, *Int-6* EP-t to +30min, *Int-7* EP-t to +60min, *Int-8* EP-t to +120min; a,b *P*<0.005.  *n* number, *RA* respiratory arrhythmia, *AVB* atrio ventricular block of 2^th^degree, *PVC* premature ventricular contraction, *ST* sinus tachycardia. Arrhythmias in int-1 (grey boxes) are not reported because they are specified in Int-2, Int-3, Int-4 that constitute it

No statistically significant differences were observed between groups at any time. A statistically significant within-group difference was observed for the RSB group between Int-2 [from − 120 min pre-surgery to induction (IND-t)] and Int-3 [from IND-t to the end of the procedure (EP-t)] (99.9 ± 21.0 vs. 90.7 ± 22.0 bpm ± SD, *P* < 0.005).

The type and frequency of cardiac dysrhythmias are reported in Table 1. Briefly, except for one animal belonging to the RSB group, all the others showed at least one arrhythmia during the recording periods (13 of 14 calves). No difference was found between groups in the time spent in arrhythmias.

### Serum cortisol concentrations

**Fig. 1:**
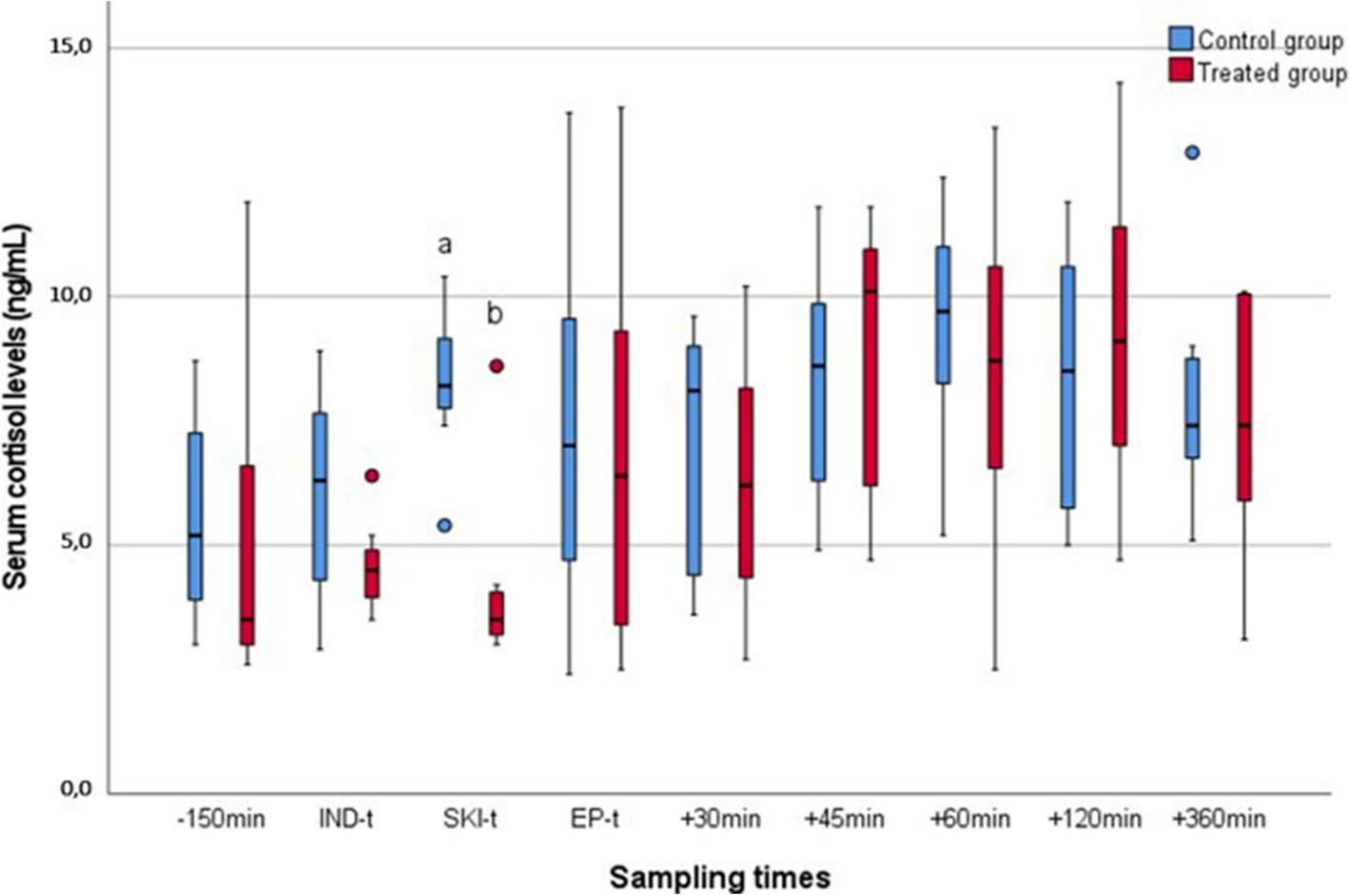
Box plot representing the median and interquartile range serum cortisol concentrations detected in 14 calves receiving ultrasound-guided rectus sheath block (RSB) or sham injection (CG) at different time intervals. Ng = nanograms; mL = millilitres; min = minutes; -150min = 150 minutes pre-surgery; IND-t = induction time; SKI-t = skin incision time; EP-t = end of surgical procedures time, +30min = 30 minutes post-surgery; +45min = 45 minutes post-surgery; +60min = 60 minutes post-surgery; +120min = 120 minutes post-surgery; +360min = 360 minutes post-surgery; a,b *P *= 0.003

 The median and interquartile range serum cortisol concentrations in groups RSB and CG and the mean ± SD concentrations observed at the different time intervals are reported in Fig. 1; Table 2, respectively. Briefly, a significant difference was observed between the RSB group and CG at skin incision (SKI-t) (*P* = 0.003, Fig. 1). An intra-group difference was found within the RSB group between IND-t and 45 min post-surgery (*P =* 0.004) and between SKI-t and 45 min post-surgery (*P* = 0.005). No statistical intra-group differences were instead observed for CG (Table 2).

**Table 2:** Average serum cortisol levels detected in calves receiving (group receiving rectus sheath block, RSB) and without receiving ultrasound-guided RSB (control group, CG) at different time-intervals

	*Serum cortisol levels*																	
	*-150 min*		*IND-t*		*SKI-t*		*EP-t*		*+ 30 min*		*+ 45 min*		*+ 60 min*		*+ 120 min*		*+ 360 min*	
	*ng/mL* ± *SD*		*ng/mL* ± *SD*		*ng/mL* ± *SD*		*ng/mL* ± *SD*		*ng/mL* ± *SD*		*ng/mL* ± *SD*		*ng/mL* ± *SD*		*ng/mL* ± *SD*		*ng/mL* ± *SD*	
***RSB***	5.3	0.14	4.6^c^	0.03	4.5^a,e^	0.08	6.8	0.15	6.2	0.11	8.9^d,f^	0.10	8.5	0.13	9.5	0.13	6.8	0.09
***CG***	5.6	0.08	6.0	0.08	8.2^b^	0.06	7.3	0.15	6.8	0.09	8.2	0.09	9.4	0.09	8.3	0.11	8.0	0.09

*ng *nanograms, *mL *millilitres, *min *minutes; *-150 min *150 min pre-surgery, *IND-t *induction time, *SKI-t *skin incision, *EP-t *end of surgical procedures, *+ 30 min *30 min post-surgery, *+45 min *45 min post-surgery, *+60 min *60 min post-surgery, *+120 min *120 min post-surgery, *+360 min *360 min post-surgery; a,b*P =* 0.003; c,d*P =* 0.004; e,f*P =* 0.005

### Unidimensional pain scale

The median [interquartile range (Q1, Q3)] UNESP-Botucatu pain score (PS) at baseline was 0 (0, 0) in both groups. Sedation significantly impacted the outcome (*P* < 0.05) and was therefore included in the final regression model as a confounding factor. The final multivariable regression model was significant (*P* < 0.01). When considering all variables in the model, pain scores were overall significantly higher than the baseline for the entire study period (*P* < 0.01). There was a significant interaction between treatment and time. Calves in the RSB group recorded lower pain scores from 45 to 120 min after recovery and at 240 min after recovery (*P* < 0.05) (Table 3).

**Table 3:** The median [interquartile range (Q1, Q3)] pain scores at 45, 60, 120 and 240 minutes after the end of the procedure (EP-t) 14 calves receiving ultrasound-guided rectus sheath block (RSB) or sham injection (CG) at different time intervals. A statistically significant difference was found in all the times considered (*P *<  0.05)

*Pain Score*							
*RSB*				*CG*			
*Times*	*MEDIAN*	*Q1*	*Q3*	*Times*	*MEDIAN*	*Q1*	*Q3*
***45 min***	1	0	3	***45 min***	4	1	6
***60 min***	1	0	3	***60 min***	3	1	6
***120 min***	0	0	1	***120 min***	1	1	2
***240 min***	0	0	1	***240 min***	1.5	0	2

*Q1* first quartile, *Q3* third quartile, *EP-t* end of surgical procedures, *min* minute

## Discussion

To the authors’ knowledge, the current study is the first to describe the clinical effects of a novel local anesthetic block for herniorrhaphy in calves. Estimating the effectiveness of analgesic actions during routine clinical procedures under field conditions represents a stimulating challenge in cattle medicine, where the continuous necessity to improve the animal health and welfare of ruminants during their entire life cycle leads to eagerness for new information and updates. The minimum standard for calves’ protection is clearly defined by the European Council Directive 2008/119/EC [18]. Annex I at point 6 states: “Any calf which appears to be ill or injured must be treated appropriately without delay, and veterinary advice must be obtained as soon as possible for any calf which is not responding to the stock keeper’s care.“ In addition, more generally, their primary safeguard falls under the provisions of Council Directive 1998/58/EC [19], also known as the “General Farm Animals Directive,“ where the third article declares: “The Member States shall make provision to ensure that the owners or keepers take all reasonable steps to ensure the welfare of animals under their care and to ensure that those animals are not caused any unnecessary pain, suffering or injury.“ Despite the detection and relief of pain gaining considerable attention in farm animals, Hartnack et al. [1] recently highlighted that only a few clinical studies were performed in cattle to assess the effect of analgesia on biological functions after abdominal surgery. In our specific case, to fulfill this ambitious aim, the authors examined the effect of RSB on the heart rate, SC and behavioral changes in calves in field conditions.

The assessment of discomfort in ruminants represents a challenge for clinicians because they naturally tend not to express pain to avoid appearing vulnerable [15]. As suggested by previous studies [7], the use of a digital Holter for scientific purposes has several advantages in this regard: (i) it provides continuous monitoring of HR and cardiac rhythm, leaving the patient in its environment; (ii) it allows the animal to behave spontaneously; (iii) it rules out the possible effects due to investigators presence during the recording. As widely known, pain stimulus is often associated with physiological, behavioral, and neuroendocrine changes [12, 14, 20]. Indeed, after the release of pro-inflammatory cytokines and the consequent activation of the hypothalamus-pituitary-adrenal axis, the sympathetic nervous system causes the release of ACTH and catecholamines [12]. An increase in myocardial activity and peripheral vasoconstriction is raised by this neuroendocrine activity, followed by a higher oxygen demand and an increase in HR [12, 20]. This study observed a significantly lower HR for the treated animals during the third period (from the IND-t to the EP-t) than the presurgical period (*P* = 0.005; Table 1). The findings might support the hypothesis that an adequate volume of bupivacaine and dexmedetomidine into the plane between the rectus abdominis muscle and its internal sheath would provide sufficient antinociception for umbilical incisions evidenced by some beneficial effects on the HR. In this regard, it might be interesting to explore the role of dexmedetomidine in more detail. Effects on the cardiac dynamic in dairy calves were previously explored by Cagnardi et al. [21], who demonstrated that injecting 5 µg/kg dexmedetomidine intravenously (IV) resulted in HR reduction. In our case, the authors chose to add 1 µg/mL as an adjuvant to the local anesthetic mixture to improve the overall analgesic effects as performed in other species [22–24]. Nevertheless, systemic absorption of this drug cannot be excluded entirely, as well as a systemic effect.

Continuing to analyze the relationship between the analgesic protocol and the cardiac dynamic, the role of the systemic administration of xylazine and ketamine should also be noted. This protocol is commonly used to produce chemical restraint in ruminants and induce sedation, dissociative anesthesia, analgesia, muscle relaxation, and lateral recumbency [25]. Nevertheless, due to the action of the α-2 adrenergic agonist on the central and autonomic nervous systems, several cardiac effects (including arrhythmias, transient initial arterial hypertension followed by hypotension, and decreased cardiac output) were associated with their use in ruminants [26, 27]. The mentioned effects, along with the prolonged food deprivation undergone by the study population, might be considered the rational explanation for the similar type and number of cardiac dysrhythmias observed in both groups [26, 28].

The measurement of serum cortisol concentrations can be considered one of the most reliable neuroendocrine parameters to evaluate the welfare of farm animals [20, 29, 30]. However, it can be profoundly influenced by stress, pain, or fear [8, 31, 32]. In this study, the RSB group expressed lower values than CG at SKI-t (*P* = 0.003; Fig. 1). The antinociceptive effects seem to be confirmed at least during the administration of the RSB (15 min pre-surgery) and the SKI-t. In contrast, the effects disappear from EP-t onwards (Fig. 1; Table 2). The nature and role of serum cortisol support the explanation. Both groups received several clinical procedures eliciting discomfort and fear from the EP-t onwards. The unusual situation, the handling, and the investigators’ presence might have influenced the outcomes and reduced the SC differences after surgery despite the preventive measures used [8, 20]. The PS results may even confirm the last deduction. As mentioned before, observation of behavioral change (e.g., abnormal standing time, lying time or posture, reduced walking time) is commonly associated with clinical triage monitoring (i.e., respiratory rate, HR and temperature) to assess health and welfare in the field [3, 33, 34]. In our study, calves receiving RSB had a lower PS values at 45 min, 60 min, 120 min, and 240 min after surgery (*P* < 0.05). The results of this clinical visual procedure may support the hypothesis of a lower perception of pain in the RSB group regardless of the SC observed. The authors ascribed these consequences to the long-term analgesic effects of the local anesthetic mixture employed [35]. Indeed, compared to lidocaine (characterized by a time-limited effect of 20–40 min), bupivacaine is characterized by a higher pKa than lidocaine and a longer duration of action (5–8 h), likely even boosted using dexmedetomidine [36, 37].

Based on these results, it appears that using a single parameter to assess the analgesic efficacy of the RSB could be inadequate because, when taken individually, there was no difference between the study groups. Instead, the simultaneous use of different assessment techniques provided additional information to explore the impact on biological functions of the ultrasound-guided RSB. Indeed, the multiparametric approach reduced the unavoidable influence of external factors (e.g., environment, investigators’ presence), increasing the accuracy of the diagnostic process as reported in the literature [16]. On the one hand, the HR and SC revealed greater reliability in detecting short-term beneficial effects during surgical procedures when the patient was unconscious and no interactions with them were present. On the other hand, the PS highlighted positive and long-term differences in the group receiving the block; in our study protocol, the visual assessment of animal behavior confirmed its reliability in measuring post-surgical pain relief when the patient began a conscious interaction [3, 11, 21].

Although the study allows attractive clinical deductions, further investigations should be performed to exceed some limitations, like a larger study population and a different study environment aiming to reduce the negative interaction of patient-environment-investigators. If the Holter and PS assessments had the advantage of minimizing the interaction with the investigators, the same cannot be said for the blood sampling that required the animal holding. The authors managed this adverse effect (i) by excluding from the analysis the short time frame of the cardiac dynamic related to this procedure and (ii) by scoring the animals with the pain scale immediately before the collection. Nevertheless, using different or less invasive diagnostic procedures might improve the quality of the results, giving further proof of the welfare-promoting properties of this locoregional anesthetic procedure.

## Conclusions

The study aimed to assess the usefulness of a clinical multiparametric approach to evaluate the effects of a novel locoregional anesthetic technique on the health and welfare of calves undergoing herniorrhaphy. Overall, the multiparametric approach allowed us to define the effects of the novel technique on animals’ health and welfare. Of all the diagnostic techniques used, the heart rate and serum cortisol concentrations showed the RSB’s beneficial effects mainly during the surgical procedure because both seem to be influenced by the conscious interaction of the patients with external factors (e.g., environment, investigator presence). On the contrary, the UNESP-Botucatu pain scale proved long-term positive effects of the technique, mainly when patients could express their natural behavior after surgery. According to our results, the ultrasound-guided RSB revealed worthy beneficial effects on animal health and welfare, justifying further studies to confirm the current findings and promote the widespread of the technique in bovine medicine.

## Materials and methods

### General

This prospective, blinded clinical trial involved fourteen calves referred for umbilical hernia repair at the Didactic Clinical Mobile Service, part of the Veterinary Teaching Hospital in the Department of Veterinary Medicine and Animal Production at the University of Naples, Federico II. The study was conducted between May 2018 and June 2020. A detailed description of the clinical procedures and sampling methods can be found in a prior study from the same research group. The earlier study aimed to introduce the use of ultrasound-guided RSB in freshly deceased calf cadavers and to assess postoperative pain in live calves undergoing herniorrhaphy under general anesthesia in field conditions [38].

### Study designs and population

Based on Lomax and Windsor [39], a minimum of five calves per group would have been required with an assumed alpha error of 0.05 and a power of 0.80. To account for potential data loss and ensure robust results, seven calves per group were included in the study. The study population was selected from a pool of 48 calves referred for suspected disorders related to the umbilical region based on specific eligibility criteria: (i) overall good health, as determined by historical data (i.e., no other health problems from birth to the surgery time); (ii) absence of any concurrent diseases at the time of enrollment, confirmed through a thorough veterinary clinical examination [40] and hematological and biochemical investigations conducted the day prior to the study [41]. Moreover, all the selected calves met the following criteria: (iii) they had a reducible umbilical hernia, which could potentially contain different types of contents within the hernia sac, such as the greater omentum, abomasum, small intestine, or large intestine; (iv) it was determined that spontaneous resolution of the hernia was unlikely, guided by the criteria provided by Weaver et al. [36] (i.e., three to six months aged, with hernia still present, possibly enlarging tendency).

### Clinical assessment and ultrasonography

Twenty-four hours before surgery, an expert clinician examined the umbilical region according to the guidelines provided by Weaver et al. [36]. Briefly, the clinician palpated the swelling and the rest of the ventral abdominal wall to make a preliminary diagnosis of an umbilical hernia and rule out any signs of pain or structures that could indicate intra-abdominal complications such as strangulation, incarceration, or sepsis. Following the physical examination, an ultrasonographic examination (MyLab™Alpha Ultrasound, ESAOTE® SpA, Genoa, IT) further investigated the hernia sac’s contents and confirmed the absence of intra-abdominal complications. The ultrasound examination was conducted with the animal in a standing position after the ventral abdominal area and the right hypochondrium were clipped and shaved, as recommended by Borriello et al. [42]. The examination involved scanning the extra-abdominal umbilical structures using both transversal and longitudinal scanning techniques. Subsequently, the intra-abdominal structures, both in front and behind the hernia sac, were examined, along with an evaluation of the liver. Convex and linear probes with frequencies ranging from 3.5 MHz to 12 MHz were utilized as necessary during the ultrasound examination to obtain detailed and comprehensive information [6].

### Rectus sheath block and surgical procedures

Calves were premedicated with xylazine 0.02–0.05 mg/kg (Nerfasin® 100 mg mL-1; Ati S.r.l., Italy) and butorphanol 0.02 mg/kg (Alvegesic® 10 mg mL-1; Dechra Veterinary Products S.r.l., Italy) intravenously (IV). Anesthesia was induced with ketamine 2.5 mg/kg IV (Lobotor®100 mg mL-1; ACME S.r.l., Italy). Before surgery, calves were assigned to either the RSB group or the CG by a random number generator (www.randomizer.org). The RSB group received a bilateral rectus sheath injection with 0.3 mL/kg of bupivacaine 0.25% (Bupivacaine Recordati; Recordati SpA, Italy) containing 0.15 µg/kg of dexmedetomidine (Dexdomitor® 0.5 mg/mL; Zoetis inc., USA), as an adjuvant to prolong the effect of the local anesthetic [24, 38, 43]. The CG received a sham injection with an equivalent volume of sterile saline (0.9% NaCl). All injections were performed by the same operator using the technique described by Ferreira et al. [17] 15 min before skin incision. A linear probe (8–13 MHz) connected to a portable ultrasound device (MyLab™Alpha Ultrasound, ESAOTE® SpA, Genoa, IT) was used to inject the solutions between the *rectus abdominis* muscle and its internal sheath, using an in-plane technique.

A conventional surgical method was used for the herniorrhaphy [44]. All procedures were performed by the same expert veterinary surgeon. The patients were placed in dorsal recumbency; the abdominal wall from the xiphoid cartilage to the pubis was clipped and washed with surgical soap (Golmar Italia SpA, Borgaretto, Italy) and tap water. Surgical scrubbing was performed with povidone-iodine solution (Betadine®, Meda Pharma SpA, Milano, Italy) and 70% alcohol repeated three times. Large sterile drapes were placed to define the surgical site. The wall defect was exposed through an elliptical skin incision and a blunt dissection of subcutaneous tissues. The hernial ring edge was bluntly dissected from surrounding tissues and completely exposed, leaving intact the hernial sac. The internal hernial sac was gently replaced into the abdomen without penetrating the peritoneum, and a “closed herniorrhaphy” was performed. A simple interrupted pattern in absorbable material (Vycril USP 2 Ethicon Vicryl™, Alcyon SpA, Cherasco, Italy) was placed and progressively closed, avoiding extensive suture tension and the insertion of underlying tissues. The subcutaneous tissues and the skin were opposed in routine manners.

If the calves responded to surgical stimulation, additional boluses of IV ketamine (0.5 mg/kg) or xylazine (0.01 mg/kg) were administered. Each calf received 1.1 mg/kg of flunixin meglumine IV (Alivios®; Fatro S.p.a., Bologna, Italy) at the end of skin closure (EP-t). Animals also received antibiotic treatment at the discretion of the referring veterinarian. The timeline of the study is detailed in Table 4.

**Table 4:**
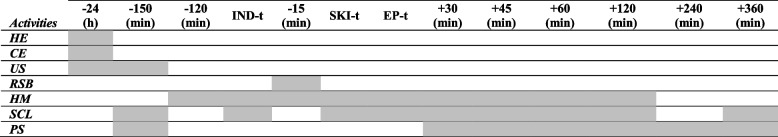
Gantt chart summarising all clinical activities carried out at different time intervals in 14 calves receiving ultrasound-guided rectus sheath block (RSB) or sham injection (CG)

*h *hours, *min *minutes, *IND-t*  induction time, *SKI-t *skin incision time, *EP-t *end of surgical procedures, *HE *hematologic evaluation, *CE *clinical examination, *US  *ultrasound, *RSB *rectal sheath block, *HM *Holter monitoring, *SCL *serum cortisol level, *PS *pain score

### Evaluation of calves’ health and welfare

Calves were isolated in a separate pen, without the possibility of interaction with people or other animals, 24 h before and after surgery to minimize the adverse effects related to their presence. Food was withdrawn for 24 h, while water for 12 h prior to induction. Approximately 150 min before induction (-150 min), another ultrasonographic examination was performed to confirm previous findings. Blood samples were collected from the jugular vein to measure pre-anesthetic serum cortisol concentrations. Twenty-four hours after the herniorrhaphy procedure, all calves underwent a clinical examination to assess their overall health status and the condition of the surgical site. Additionally, the farmers received follow-up phone calls 15 days after the surgery.

### Heart Rate and Rhythm

For each calf, four hours of continuous Holter monitoring (120 min before surgery and 120 min after surgery) were performed following the procedure suggested by Guccione et al., [45] and based on the use of a 3-channel digital Holter recorder (modelVX3i series C; Biomedical Systems, Brussels, Belgium). The left and right chests were clipped cleaned with alcohol where the electrodes were to be placed (Euro ECG electrodes; Fiab, Vicchio, Italy). The electrodes were connected to the respective leads from the receiver (placed on the middle third of the left thorax). For leads (L) 1 and 2, the respective positive electrodes were positioned at the level of the fifth and sixth left intercostal spaces (ICS) behind the olecranon. The negative electrode was positioned over the left side of the thorax behind the withers, while the ground one was placed over the left mid-thorax between the other two electrodes. For L3, the positive electrode was positioned on the seventh left ICS, 2 inches above the other two positive electrodes. The negative electrode was applied in the same position but on the right side. A final bandage with cotton and an elastic band was used to protect the equipment.

Data obtained were stored on a flash memory card (2 Mb CompactFlash; Delkin Devices, Taipei City, Taipei, Taiwan), downloaded, and analyzed by an expert veterinary cardiologist using dedicated software (Holter Scanner Century model C 3000; Biomedical Systems, Maryland Heights, USA). The software was designed for human patients; therefore, the recordings were manually analyzed. The software gave HR only once the artifacts were manually removed. Nevertheless, a manual calculation of ventricular HR was also performed to confirm the data provided by the device, as suggested by Guccione et al. [45] (i.e., counting R-R intervals in 6 s and multiplying the results by 10). The time intervals associated with the interaction with the animals during blood samplings, starting from the investigator’s entry into the separate pen and ending with his departure, were documented in a notebook. These time intervals were subsequently excluded from the analysis to ensure the analysis was unaffected by the duration of the human-animal interaction. Overall, the HR was manually calculated 48 times per calf (every 5 min). The presence of cardiac dysrhythmias was classified according to McGuirk [46].

### Blood sampling procedures and testing

A jugular blood sample was obtained at -150 min to establish baseline SC. After the first sample, trichotomy and surgical scrub of the jugular region were performed to place an intravenous catheter (14 Gauge, L/A long-term catheter, Anicath, Millipledge, UK) [47]. Subsequent samples were collected through the catheter, following the protocol described by Cagnardi et al. [21]. Before each collection, 5 mL of blood was drawn, and the catheter was flushed with 10 mL of NaCl 0.9%.

Blood samples for cortisol measurement were collected at IND-t, SKI-t, EP-t, then at 30 min, 45 min, 60 min, 120 min, and 360 min after surgery. The sampling procedure was performed immediately after the behavioral scoring when the two actions coincided (Table 4). The samples were then placed into serum tubes (Vacutainer, Becton and Dickinson, Franklin Lakes, US) and centrifuged at 908 g for 15 min to separate the serum. Serum cortisol concentration was quantified using a solid-phase competitive chemiluminescent enzyme immunoassay (Bovine Cor Elisa Kit, Fine Biotech Co., Ltd, Wuhan, China) following the manufacturer’s instructions [21]. An automated analyzer system was used as the reader (Siris S EIA Reader, RADIM SEAC, Next Level s.r.l. Calenzano, Italy). The assay’s calibration range was 0.391-25 ng/mL, while the analytical sensitivity was 0.234 ng/ml.

### Unidimensional pain scale

All calves were scored with a unidimensional, composite pain scale to quantify the discomfort after the surgical procedures [3, 15]. The UNESP-Botucatu scale is described in detail in Additional file 1. In summary, this scale involved monitoring five behavioral changes (1°: locomotion; 2°: interactive behavior; 3°: activity; 4°: appetite; and 5°: miscellaneous behaviors) using an ordinal scale that ranged from 0 (indicating normal behavior) to 2 (indicating complete abnormality), with each parameter being assessed. The scores for these five parameters were summed, resulting in a maximum score of 10 points per animal. The calves were evaluated 150 min before surgery (-150 min), 30 min, 45 min, 60 min, 120 min, 240 min, 360 min after EP-t. Animals were observed continuously for 5 min. An experienced researcher, knowledgeable in bovine behavior and welfare and blinded to the treatment, entered the animals’ pen approximately 25 min before the evaluation to allow the animals to adapt to their presence (except for the scorings 30 min, 45 min, and 60 min in which the investigator wasto continuously present).

### Statistics

Data were expressed as absolute numbers, percentages, median [interquartile range (Q1, Q3)] or mean ± SD (standard deviation). Variables were analyzed by standard descriptive statistics. Normality was assessed using the Shapiro-Wilk normality test, normal probability plots, and histograms. Holter monitoring data were divided into eight arbitrarily defined time intervals (Int-1 = 120 min pre-surgery to 120 min post-surgery; Int-2 = 120 min pre-surgery to IND-t; Int-3 = IND-t to EP-t; Int-4 = EP-t to 120 min post-surgery; Int-5 = EP-t to 15 min, Int-6 = EP-t to 30 min, Int-7 = EP-t to 60 min, Int-8 = EP-t to 120 min) for analysis. Heart rate and SC were compared between groups at different time points using the Two-Tailed Students’ t-test for independent means and intra-group comparisons using one-way repeated measures ANOVA with Bonferroni correction. Difference between the expected and the observed frequencies in some categories of data (i.e., overall minutes of respiratory arrhythmia or sinus tachycardia/overall minutes of recording, overall number of premature ventricular contractions or 2nd -degree atrioventricular blocks /overall number of beats) were compared using contingency tables, *χ*^2^-test or Fisher’s exact test as appropriate. An alpha level < 0.05 was considered statistically significant, while the Bonferroni correction set a new alpha value = 0.006 for the multiple comparisons.

Baseline PS (-150 min) between calves receiving RSB and CG were compared using the Mann–Whitney U test. The median difference (95% confidence interval) between calves in the two treatment groups was calculated using the Hodges-Lehman method proposed by Conroy [48] for nonparametric data. Data were then rank-transformed for further analysis [49]. The differences in pain scores between treatment groups over time were evaluated using a mixed-effect linear regression model, with the calf as the random effect and time and treatment as fixed effects. An interaction term between time and treatment was also included. Variables significantly impacting the outcome (*P* < 0.05) were retained in the model.

All statistical data were analyzed using dedicated software (SPSS, Version 27.0.0, Chicago, IL, USA).

### Supplementary Information

**Additional file 1: Table A. **UNESP-Botucatu unidimensional pain scale used in calves receiving and without receiving ultrasound-guided RSB at different time intervals.

## Data Availability

The datasets are available from the corresponding author upon reasonable request.

## Abbreviations

AVB: Atrio ventricular block of 2th degree
bpm: Beats per minute
CE: Clinical examination
CG: Control group
CI: Confidence intervals
EP-t: End of surgical procedure
HR: Heart rate
H: Hours
HE: Hematologic evaluation
HM: Holter monitoring
ICS: Intercostal spaces
IND-t: Induction time
Int: Intervals
L: Leads
min: Minutes
n: Number
SC: Serum cortisol
SKI-t: Skin incision time
PS: Pain score
PVC: Premature ventricular contraction
RSB: Rectus sheath block
RA: Respiratory arrhythmia
SD: Standard deviation
ST: Sinus tachycardia
US: Ultrasound
