# Supplementary material for: Cardiovascular changes, laboratory findings and pain scores in calves undergoing ultrasonography-guided bilateral rectus sheath block before herniorrhaphy: a prospective randomized clinical trial
Source: BMC Vet Res. 2023 Oct 5;19:191. doi: 10.1186/s12917-023-03754-6 (PMC10552199; doi:10.1186/s12917-023-03754-6)
Supplement: Supplementary file 1 — Additional file 1: Table A. UNESP-Botucatu unidimensional pain scale used in calves receiving and without receiving ultrasound-guided RSB at different time intervals. [file 12917_2023_3754_MOESM1_ESM.docx]

**TITLE:**

**Cardiovascular changes, laboratory findings and pain scores in calves undergoing ultrasonography-guided bilateral rectus sheath block before herniorrhaphy: a prospective randomized clinical trial.**

**M. C. Alterisio*, F. Micieli*, G. Della Valle*, L. Chiavaccini**^†^**, G. Vesce*, P. Ciaramella*^1^, and J. Guccione***

***** Department of Veterinary Medicine and Animal Productions, University of Napoli “Federico II,” Via Delpino 1, 80137, Napoli, Italy**.**

^†^ **Anesthesiology and Pain Management, Department of Comparative, Diagnostic, and Population Medicine, College of Veterinary Medicine, University of Florida, 2015 SW 16^th^ Ave, Gainesville, 32608, Florida, Unites States of America.**

**^1^ Corresponding author:**

Prof. Paolo Ciaramella

**Department of Veterinary Medicine and Animal Productions,**

**University of Napoli Federico II, Napoli, Italy.**

**Address: Via Delpino 1, 81100, Caserta (Italy)**

**Email:** [paociara@unina.it](mailto:paociara@unina.it)

**Telephone: 0039 0812536011**

**Table A.** UNESP-Botucatu unidimensional pain scale used in calves receiving and without receiving ultrasound-guided RSB at different time-intervals.

| **Parameters** | **Score/Criterion** |
| --- | --- |
| **Locomotion** | (0) Walking with no obviously abnormal gait  (1) Walking with restriction, ma be with hunched back and/or short steps  (2) Reluctant to stand up, standing up with difficulty or not walking |
| **Interactive behaviour** | (0) Active: attention to tactile and/or visual and/or audible environmental stimuli; when near other animals, can interact with and/or accompany the group  (1) Apathetic: may remain close to other animals, but interacts little when stimulated  (2) Move less frequently in the pasture or only when stimulated |
| **Activity** | (0) Moves normally  (1) Restless, moves more than normal or lies down and stands up with frequency  (2) Move less frequently in the pasture or only when stimulated |
| **Appetite** | (0) Normorexia and/or rumination  (1) Hyporexia  (2) Anorexia |
| **Miscellaneous behaviours** | - Wagging the tail abruptly and repeatedly - Licking the surgical wound - Moves and arches the back when in standing posture - Kicking/foot stamping - Hind limbs extended caudally when in standing posture - Head below the line of spinal column - Lying down in ventral recumbency with full or partial extension of one or both hind limbs. - Lying down with the head on/close to the ground - Extends the neck and body forward when lying in ventral recumbency   (0) All of above-described behaviours are absent  (1) Presence of 1 of the behaviours described above  (2) Presence of 2 or more of the behaviours described above |
